# A simple and robust method for isolating and analyzing chromatin-bound RNAs in Arabidopsis

**DOI:** 10.1186/s13007-022-00967-y

**Published:** 2022-12-12

**Authors:** Qiqi Zhang, Fengli Zhao, Zhe Wu, Danling Zhu

**Affiliations:** 1 Harbin Institute of Technology, Harbin, 150001 China; 2 Key Laboratory of Molecular Design for Plant Cell Factory of Guangdong Higher Education Institutes, Institute of Plant and Food Research, Department of Biology, School of Life Sciences, Southern University of Science and Technology, Shenzhen, 518055 China

**Keywords:** Chromatin-bound RNAs (CB-RNAs), Transcription regulation, Co-transcriptional splicing (CTS), *Arabidopsis thaliana*

## Abstract

**Background:**

Chromatin-bound RNAs are the primary product of transcription that undergo on-chromatin processing such as capping, splicing, and polyadenylation. These processing steps then determine the fate of RNAs. Albeit its vital importance, a simple and robust method for isolating different fractions of chromatin-bound RNAs is missing in plants.

**Result:**

Here, we describe our updated method and the associated step-by-step protocol for chromatin-bound RNAs isolation in *A. thaliana*. The chromatin-bound RNAs isolation is based on the 1 M UREA wash that removes the majority of non-chromatin-associated proteins from the nucleus, as previously developed in mammalian cells. On-demand, the isolated chromatin-bound RNAs can be either used directly for gene-specific analysis or subject to further rRNA removal and also the optional polyadenylated RNA removal, followed by high-throughput sequencing. Detailed protocols for these procedures are also provided. Comparison of sequencing results of chromatin-bound RNAs with and without polyadenylated RNA removal revealed that a small fraction of CB-RNAs is polyadenylated but not yet fully spliced, representing RNA-processing intermediate on-chromatin.

**Conclusion:**

This optimized chromatin-bound RNAs purification method is simple and robust and can be used to study transcription and its-coupled RNA processing in plants.

**Supplementary Information:**

The online version contains supplementary material available at 10.1186/s13007-022-00967-y.

## Background

As sessile organisms, plants perceive the fluctuant environmental stimuli and adjust their internal gene expression constantly to be able to adapt to the local conditions. To a certain extent, plants’ phenotypic plasticity is determined at the transcriptional level. However, transcription is a rather dynamic process; the steady-state RNA levels are an outcome of transcription initiation, productive elongation, and termination, followed by RNA processing and decay. Although conventional mRNA-seq provided a robust way to convey transcription control, it is only by directly tracking newly synthesized RNA that the instant changes in plant responses to developmental, environmental stimuli, and disease can be revealed.

Over the past decades, a series of high-throughput sequencing methods have been developed for investigating nascent RNA molecules from the pool of cellular RNA [1]. According to the different strategies used for the enrichment of nascent RNA, those methods can be classified as CB-RNA-seq (Chromatin-Bound RNA-seq), which involves the extraction of chromatin-associated RNAs by rigorous urea wash steps [2–6]; NET-seq (Native Elongating Transcript sequencing), which isolates the Pol II-associated RNAs and requires immunoprecipitation of Pol II [7, 8]; GRO-seq (Globe nuclear Run-On sequencing), which can capture the RNAs from elongation competent Pol II complex by performing a run-on with isolated nuclei [9, 10]; TT-seq (Transient Transcriptome sequencing) that involves a brief exposure of cells to the nucleoside analog 4-thiouridine (4sU), and enables the mapping of transcription active region and the estimation of RNA synthesis and degradation [11]. The techniques mentioned above were used to answer questions about transcriptional regulation on the nascent RNA level, and each has its strength and limitation. Notably, despite the widespread nascent RNA-related research in other model systems, research based on nascent RNA in plants was just emerging owing to technical difficulties.

Since 2015, the GRO-seq method has been modified and adapted to profile the nascent transcripts from Pol II and Pol V in different plant species [12–15]. Pol II dynamics in Arabidopsis seedlings were also comprehensively studied by pNET-seq, a method that employs immunoprecipitation with different antibodies targeting Pol II isoforms with different CTD phosphorylation [15]. It was shown that Pol II with different phosphorylated CTD gave distinct association patterns [15]. By plaNET-seq, a NET-seq method that employs immunoprecipitation of total Pol II from a transgenic tagged line, it was reported that the promoter-proximal Pol II stalling function to predisposing genes for transcriptional activation upon cold exposure [16]. Notably, although NET-seq and GRO-seq efficiently capture RNAs associated with elongating Pol II complex, these methods involve Pol II immunoprecipitation with high affinity Pol II antibody (NET-seq), or Br-UTP incorporation into the nascent RNA during in vitro nuclear run-on (GRO-seq). These steps dramatically increase the difficulty of operations.

We have initially adapted the CB-RNAs isolation method for the study of *FLC* in Arabidopsis [17]. Combined with mathematical modeling, the unique CB-RNA distribution pattern along *FLC* intron1 revealed autonomous pathway components such as FCA and FLD functions to coordinately repress transcription initiation and elongation [17]. Recently, by CB-RNAs isolation in combined with Illumina high-throughput sequencing, we and others showed that co-transcriptional splicing was widespread in *A. thaliana* [18, 19]. Since then, CB-RNAs isolation was considered the most straightforward approach to obtain nascent RNAs in plants and a number of studies have successfully applied this method for CB-RNAs isolation [20–22]. Compared with other approaches, isolating CB-RNAs only involves two washes using a urea-containing buffer after nuclei extraction; hence it is much more straightforward and can be combined with a gene-targeted approach, next-generation sequencing, or long-read sequencing to investigate transcription and its coupled pre-mRNA splicing. With minor modifications, CB-RNA isolation method should be applicable in a wide range of plant species as already been demonstrated in soybean [20]. Albeit the great simplicity and durability of CB-RNAs isolation, a detailed description of the principles and cautions of this method for plant is missing.

Here, we describe our up-to-date method for chromatin-bound RNAs isolation from *Arabidopsis thaliana* seedlings. Combined with quantitative PCR or next-generation sequencing, the isolated CB-RNAs is suitable for analyzing the co-transcriptional intron splicing status both at the single gene and genome-wide level. For a given locus, the CB-RNA reflects the sum of elongating RNA (RNAe: RNAs being transcribed at the locus) and full-length polyadenylated RNA (RNAf: RNAs have been fully transcribed and polyadenylated but not yet released from chromatin). Hence, we also provided a step-by-step method to separate those two CB-RNA fractions and analyzed the corresponding distinct features. Our data showed that polyadenylated RNA likely represents the intermediate of on-chromatin RNA processing and accounts for only a small fraction of CB-RNAs. We thus propose a guideline on when the rRNA and or polyadenylated RNA needs to be removed before downstream analysis. Our work provided a simple and robust CB-RNAs isolation method in Arabidopsis, which in principle can be easily adapted to be used in a wide range of plant species.

## Results

### Isolation of chromatin-bound RNAs from Arabidopsis seedlings

**Fig. 1:**
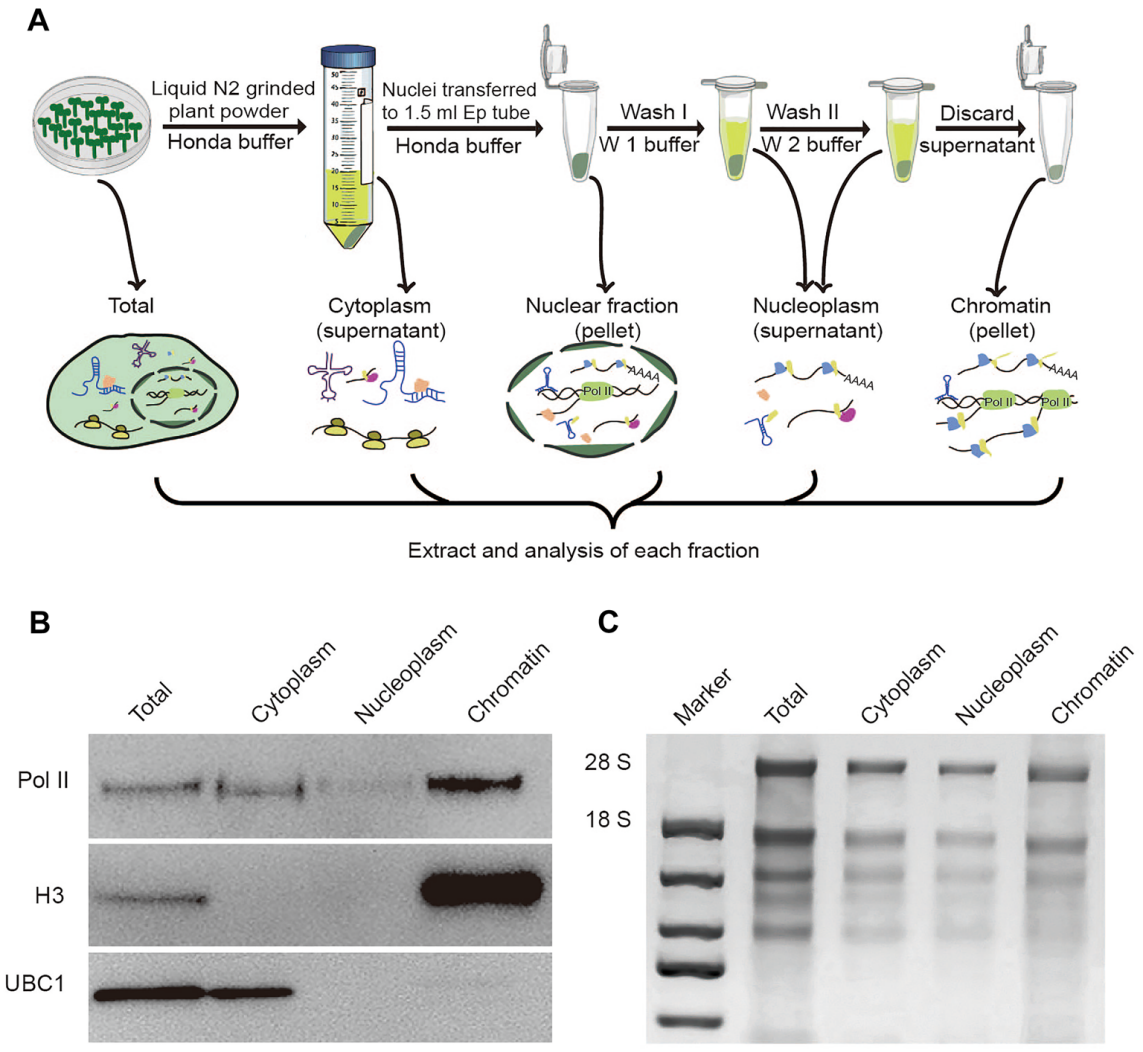
A brief workflow of chromatin-bound RNA extraction in plants. **A** The cartoon demonstrates the workflow of chromatin-bound RNA extraction in Arabidopsis. **B** Western-blot detection of different proteins in different fractions that are obtained by chromatin-bound RNA extraction. Note that the UBC1 is absent from the nucleoplasm and chromatin fraction, while H3 and Pol II are highly enriched in the chromatin fraction. **C** Gel-electrophoresis detection of RNAs in different fractions. Note that 28S and 18S rRNA bands can be observed clearly in different fractions, indicating RNA degradation is minimal during extraction

After transcription initiation, RNA polymerase II (Pol II) forms a stable complex with DNA template which resists nonionic detergent and urea. Taking advantage of this feature, Pol II associated nascent RNAs were successfully isolated from the free cytoplasmic and nucleoplasmic RNAs in different model systems, including mammals, Drosophila, and yeast. Based on these pre-existing knowledge, we describe a method suitable for CB-RNAs extraction in Arabidopsis and likely other plant species such as soybean. The extraction procedures mainly involve a nuclei extraction step followed by two stringent wash steps with a buffer containing urea (Fig. 1A). The resulting pellet was enriched with elongating nascent RNAs bound by Pol II, as well as some polyadenylated that is not yet released from chromatin. As the quality control, the protein samples from each extraction step were isolated and assessed by western blot using antibodies specific to Pol II and histone 3 (H3), which are markers of chromatin. A ubiquitin-conjugating enzyme localized in the cytoplasm, UBC1, served as another control. We found that most Pol II and H3 were detected in the chromatin fraction, while UBC1 was found mainly in the total and cytoplasm fraction (Fig. 1B), suggesting chromatin-associated protein was successfully enriched in the chromatin fraction. To check the integrity of the isolated RNAs, each fraction was checked by electrophoresis. 2% low-melted agarose gel and SYBR gold fluorescent dye were used to achieve great sensitivity and save the amount of CB-RNAs used. Notably, the 28S and 18S rRNA bands were clearly observed in chromatin bound RNAs fraction, indicating the integrity of CB-RNAs (Fig. 1C).

### Assessment of nascent RNA enrichment in the isolated CB-RNAs by qPCR

**Fig. 2:**
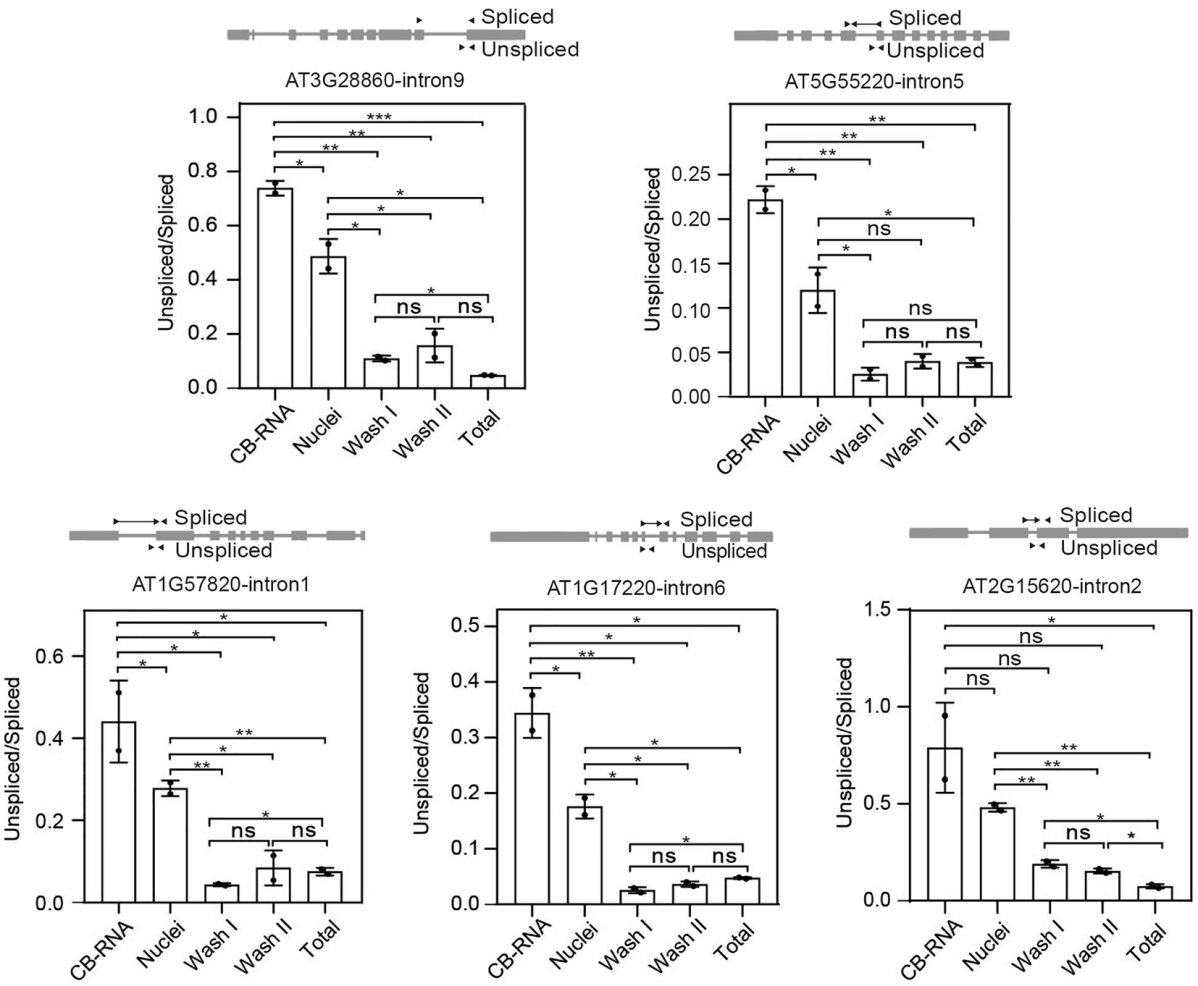
Q-PCR analysis to evaluate the enrichment of nascent RNA in different fractions.The qPCR results showed 5 specific introns’ enrichment at different RNA fractions. The Y-axis indicates the values of unspliced RNA normalized to spliced RNA. The primer locations are shown at the top of each gene structure. Note that the unspliced RNA is mostly enriched in the CB-RNA fraction. Data are presented as mean ± SEM (n = 2). Asterisk indicates significant difference among various RNA fractions based on two-tailed Student’s test

CB-RNAs isolation captures all the chromatin-associated RNA species, including the nascent transcripts containing introns that are going to be, but not yet spliced out. This allows for studying co-transcriptional processes, such as co-transcriptional intron splicing (CTS) at a single gene or in the whole genome. Here, to evaluate the enrichment of nascent RNA in different extraction fraction, the ratio of unspliced RNA to spliced RNA of a specific intron at 5 randomly selected genes were estimated (Fig. 2). Primer pairs that amplify regions correspond to intron- exon junction and exon-exon junction were designed to pick up the unspliced and spliced RNA, respectively. With these primers, the quantitative real-time PCR were then performed on different fractions that were kept during the CB-RNAs isolation procedure (Fig. 1A), that is, the nuclei fraction that was kept after nuclei extraction, the wash I fraction that resembles the nucleoplasm, the wash II fraction that contains contaminated nucleoplasm and chromatin fraction that are released by the second wash, and the CB-RNA fraction. As shown in Fig. 2, the ratio of unspliced RNA to the spliced RNA at the given exon–intron-exon unit in the wash I and wash II fractions stays at a relatively similar value to that in total RNA, suggesting the stable association between nascent transcripts and chromatin during urea washing. Importantly, the ratio of unspliced RNA to the spliced RNA is higher in the nuclei fraction compared with that in the total RNA fraction and is highest in the CB-RNA fraction, suggesting the successful enrichment of nascent RNAs.

### Workflow of sequencing library construction for CB-RNA-seq

**Fig. 3:**
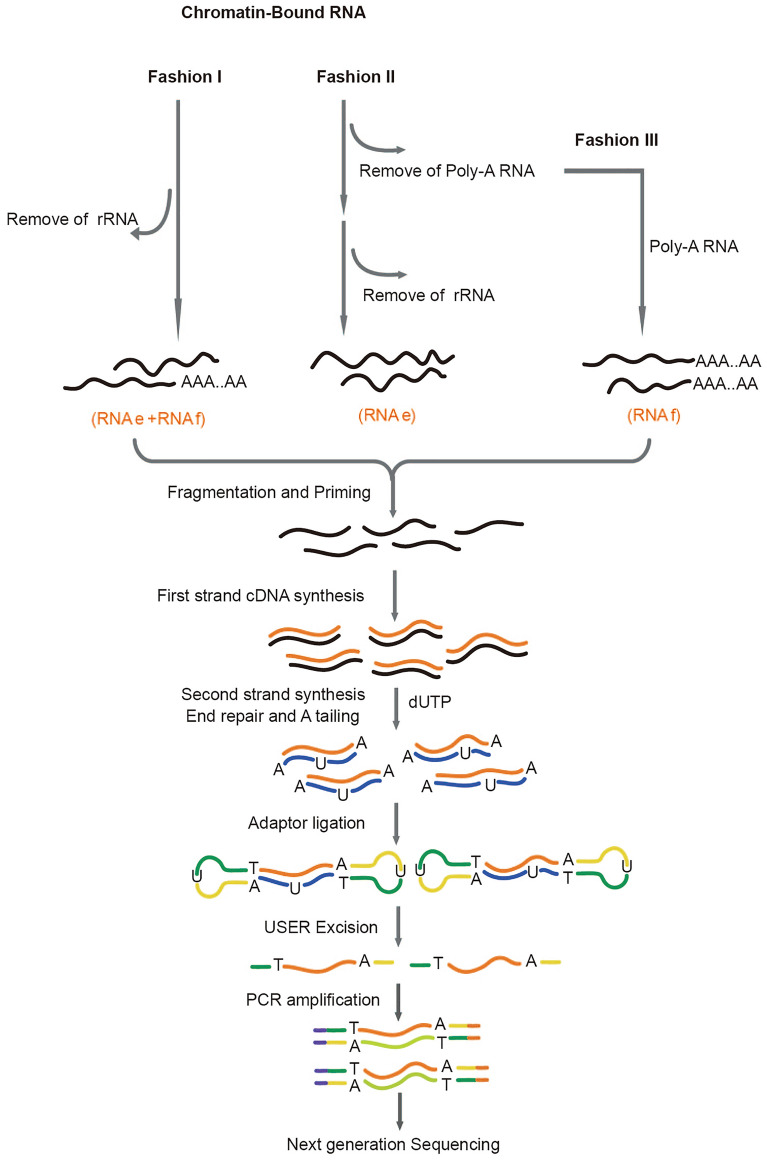
Library construction workflow of mRNA seq and CB-RNA-seq.The workflow of library construction steps is summarized. The different RNA fractions (RNAe, RNAf, RNAe + RNAf) subjected to the library construction are indicated in red. Instructions on how to isolate each RNA fraction are provided in the method section

To investigate the intron splicing status on a genome-wide scale, we performed CB-RNA-seq. As shown in Fig. 1C, ribosomal RNAs account for the majority of isolated CB-RNAs, indicating that the removal of rRNA prior to sequencing library construction is needed. Apart from the ribosomal RNAs, we reasoned that another major class within CB-RNAs is the RNAs bound by Pol II that have undergone elongation (RNAe, elongating RNAs). In addition, the isolated CB-RNAs could contain polyadenylated RNAs (RNAf, full-length RNAs) that may represent contaminated mature mRNA and or nascent RNAs that were already polyadenylated but not yet released from chromatin. To determine the relationship between RNAe and RNAf and better understand their biological relevance, we constructed CB-RNA-seq libraries in three parallel fashions (Fig. 3). In fashion I (RNAe + RNAf), the isolated CB-RNAs were subject to rRNA removal using RiboPool oligo probes that target rRNA and then used for RNA-seq library construction (Fig. 3). In the second fashion (RNAe), the CB-RNAs were subjected to polyadenylated RNA removal using oligo d(T) probes (Fig. 3). The resulting CB-RNAs were then subject to rRNA removal and followed by sequencing library construction. In the third fashion (RNAf), the chromatin-bound polyadenylated RNAs, as isolated in the fashion II, were subject to sequencing library construction (Fig. 3). For all three fashions, strand-specific sequencing library construction was performed with NEBNext Ultra II Directional RNA Library Prep Kit based on the dUTP method [23, 24] (Fig. 3). A sequencing library of the mature polyadenylated mRNA was also made as a control. The resulting strand-specific library was then subject to paired-end sequencing using an Illumina platform (Table 1).

**Table1:** NGS data summary of CB-RNA-seq and mRNA-seq

Sample name	Raw read pairs	Raw read length (bp)	Clean read number	Clean read length (bp)	Mapping read	Uniq-mapping read
mRNA-Rep1	41,856,806	12,557,041,800	72,350,924	10,457,056,137	71,225,984	67,162,647
mRNA-Rep2	47,478,548	14,243,564,400	83,689,834	12,100,412,269	82,363,749	80,335,880
mRNA-Rep3	38,940,987	11,682,296,100	67,636,210	9,773,482,296	66,554,070	65,202,996
CB-RNA-Rep1	16,969,580	5,090,874,000	17,757,542	2,628,581,958	17,275,196	16,534,432
CB-RNA-Rep2	20,996,926	6,299,077,800	20,555,956	3,043,500,976	19,909,023	19,109,633
RNAe-Rep1	6,852,120	2,055,636,000	9,807,400	1,456,490,416	8,927,603	8,211,434
RNAe -Rep2	9,497,294	2,849,188,200	13,625,772	2,024,733,454	12,487,382	11,427,750
RNAe + f-Rep1	5,779,715	1,733,914,500	7,926,760	1,175,219,966	7,208,602	6,486,167
RNAe + f-Rep2	7,359,120	2,207,736,000	8,896,990	1,319,957,238	8,110,068	7,243,658
RNAf-Rep1	30,374,722	9,112,416,600	48,166,706	7,148,469,139	47,623,079	43,707,190
RNAf-Rep2	27,261,183	8,178,354,900	44,274,638	6,571,437,414	43,750,796	41,734,703

### Nascent RNA profiling of Arabidopsis seedling by CB-RNA-seq

**Fig. 4:**
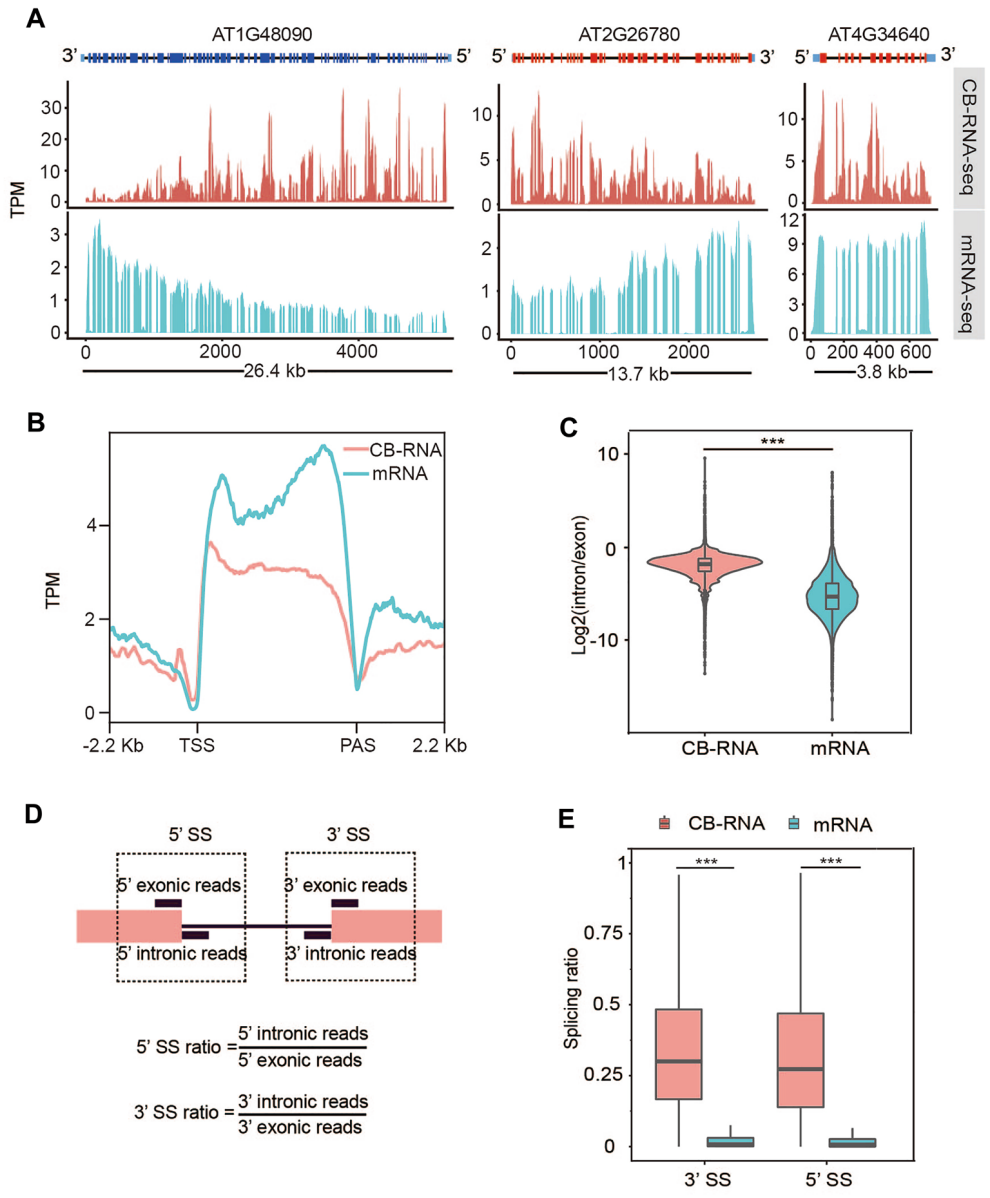
The overview of CB-RNA-seq and mRNA-seq. **A** Three examples showed CB-RNA-seq (red) and mRNA-seq (blue) results. A 5ʹ to 3ʹ declining slope is observed in the CB-RNA-seq. The gene structure is indicated at the top of each track, and the length of each gene is indicated at the bottom. **B** Meta profile showed the reads distribution of CB-RNA-seq and mRNA-seq along the gene. TSS: transcription start site. PAS: Polyadenylation site. **C** Comparison of gene’s intron/exon ratio between CB-RNA-seq and mRNA-seq. ***p < 0.001, Wilcoxon test. **D** Diagram showed the calculation method of 5ʹ SS ratio and 3ʹ SS ratio. **E** Boxplot showed splicing ratio of 5ʹ SS and 3ʹ SS ratio of CB-RNA-seq and mRNA-seq in Arabidopsis. The boxplot showed the median and the 25th and 75th percentiles

We first analyzed the sequencing reads distribution along genes of CB-RNA-seq data with only the rRNA depleted but with polyadenylated RNA kept (RNAe + RNAf). Compared with mRNA-seq data, two distinct features were observed in CB-RNA-seq data. Firstly, the read density of CB-RNA-seq declined gradually from 5ʹ to 3ʹ end, while no such pattern was observed in mRNA-seq (Fig. 4A and B). Assuming that elongating RNA dominates the CB-RNA fraction, there would be more nascent RNA at gene 5ʹ end than 3ʹ end, given RNA synthesis always occurs from 5ʹ end to 3ʹ end. Instead, a slightly opposite trend was observed in the mRNA-seq data, likely due to enrichment of RNA 3ʹ end when purifying the mRNA using oligo d(T). Secondly, as we expected, CB-RNA-seq detects much more intron reads than mRNA-seq due to the capture of nascent RNAs with intron not yet removed (Fig. 4A). Consistently, the ratio of intronic reads to exonic reads in CB-RNA-seq is much higher than that in mRNA-seq (Fig. 4C). To precisely calculate the co-transcriptional splicing (CTS) efficiency, we adopted a 3ʹSS/5ʹSS ratio to measure the extent of co-transcriptional splicing [4, 25, 26]. Briefly, at the given intron–exon boundary, we calculated the ratio of intronic reads over the adjacent exonic reads to yield a 5ʹ splicing site ratio (5ʹSS ratio) and a 3ʹ splicing site ratio (3ʹSS ratio) (Fig. 4D). Thus, the 5ʹSS/3ʹSS ratio reflect intron retention levels at the chromatin and is negatively correlated with splicing efficiency. As we expected, the median value for CB-RNA was around 0.25, which is in line with previous results [18, 19] and it was significantly higher than that in mRNA-seq (Fig. 4E), suggesting that splicing predominantly occurs at the co-transcriptional level.

### A small proportion of CB-RNAs are polyadenylated and generally represent RNA processing intermediates on chromatin.

**Fig. 5:**
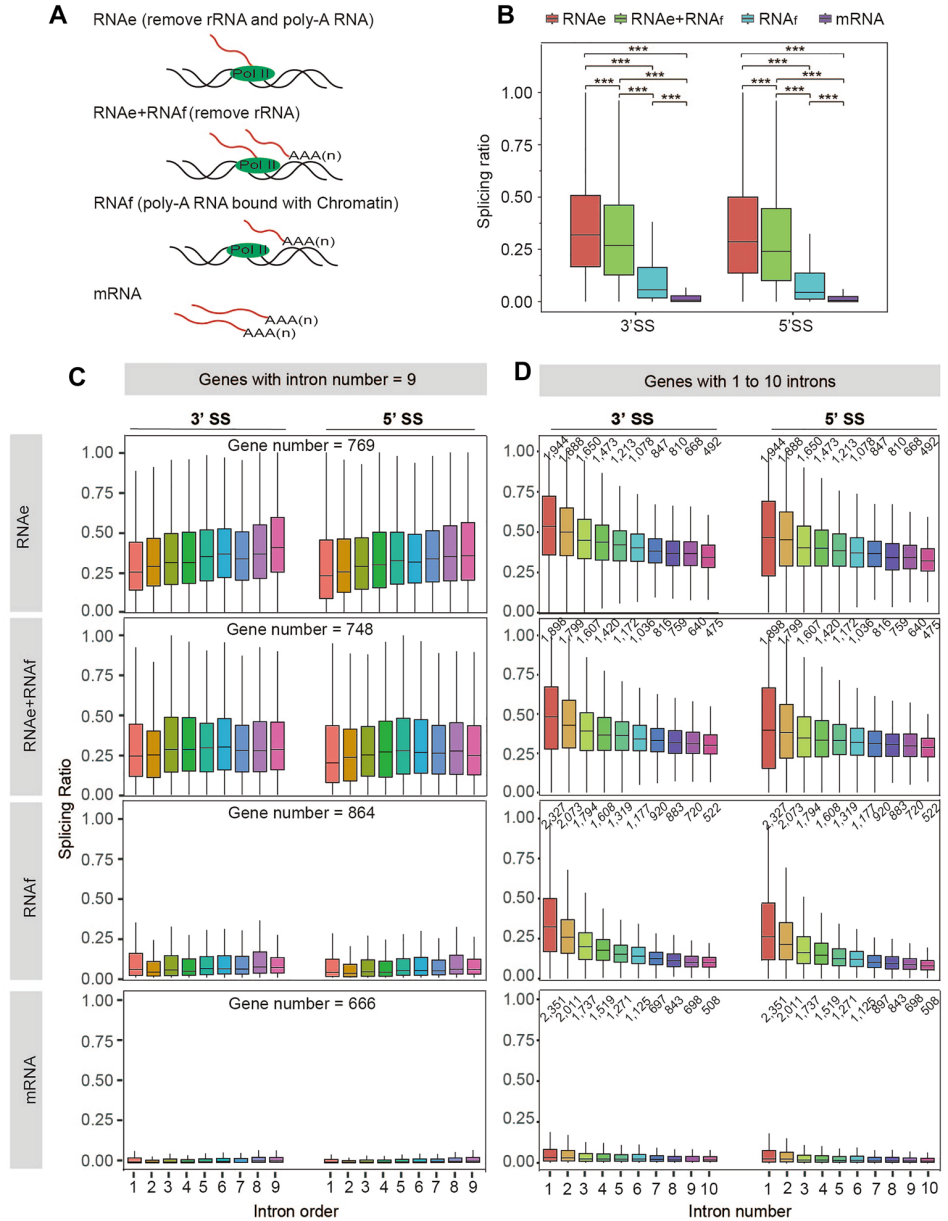
The comparison of intron splicing status in different CB-RNAs. **A** The cartoons show different fractions of the chromatin-bound RNAs. RNAe indicates the nascent being transcribed at the locus. RNAf indicates the full-length RNA, which is polyadenylated yet still attached to the chromatin. **B** Boxplot showed splicing ratio of 5ʹ SS and 3ʹ SS ratio of RNAe, RNAf, and RNAe + RNAf. ***p < 0.001, Wilcoxon test. The boxplot showed the median and the 25th and 75th percentiles. **C** The relationship between co-transcriptional splicing (CTS) and intron order in RNAe, RNAf, and RNAe + RNAf. The genes containing 9 introns were selected as representatives. X-axis indicated the intron order from 1st to 9th. The analyzed gene number was indicated at the top of each box. **D** The relationship between co-transcriptional splicing (CTS) and intron number in RNAe, RNAf, and RNAe + RNAf. The genes containing 1 to 10 introns were selected as representatives. X-axis indicated the intron number from 1 to 10. The analyzed gene number was indicated at the top of each boxplot

Next, we wondered about the relationship between RNAe and RNAf in the isolated CB-RNAs. Thus, we analyzed and compared the sequencing results obtained from RNAe + RNAf, RNAe, and RNAf (Figs. 3 and 5A). We found that the 5ʹ SS and 3ʹ SS ratio in RNAe is only slightly higher than that of RNAe + RNAf (Fig. 5B), suggesting that RNAf only accounts for a minor proportion of CB-RNAs. Indeed, the 5ʹ to 3ʹ declining pattern in RNAe + RNAf also supports the above conclusion (Fig. 5B). The SS ratio of RNAf is much lower than that of RNAe or RNAe + RNAf (Fig. 5B), suggesting that the introns in RNAf are largely removed, consistent with the fact that these RNAs are already polyadenylated. Notably, the SS ratio of RNAf is still significantly higher than that of mature mRNA (Fig. 5B), suggesting RNAf is most likely an intermediate of RNA processing on chromatin instead of the contaminated mature mRNAs during CB-RNAs isolation.

Since multiple factors could also affect CTS efficiency, such as the intron positions at a given gene and intron numbers, we looked at how CTS efficiency correlates with the order of intron within genes. We extracted genes with the same intron numbers and analyzed the corresponding 3ʹSS and 5ʹSS at each individual intron from RNAe, RNAf, and RNAe + RNAf (Fig. 5C). The results showed that both the 3ʹSS and 5ʹSS ratios were gradually increased from the first intron to the last intron (Fig. 5C, and Additional file 2: Fig. S1), consistent with a “first come, first serve” model of CTS [27]. Notably, such a trend was observed clearly in the RNAe (Fig. 5C), indicating that the removal of RNAf when performing CB-RNA-seq could be beneficial when accurate analysis of CTS is required. In addition, the absence of such a trend in RNAf and, to a certain extent, also in RNAe + f suggests that nascent RNAs could be held at chromatin for a substantial time post active elongation, enabling effective on-chromatin splicing (Fig. 5C).

Next, as intron numbers affect splicing efficiency [18], we plotted the 3ʹSS and 5ʹSS ratios with the genes containing different intron numbers. The results showed that the numbers of introns/exons negatively correlated with the average 5ʹSS and 3ʹSS ratio in all three fractions of CB-RNAs (Fig. 5D), suggesting strong cooperativity of splicing among different exons/introns within a gene. Notably, the fact that such cooperativity can also be seen in RNAf but not in mature mRNA, further strengthens the conclusion that RNAf represents RNA processing intermediates that are polyadenylated but still undergo processing steps such as RNA splicing on chromatin (Fig. 5D).

## Discussion

Nascent RNA sequencing has been widely used to study the co-transcriptional regulation in many species. However, isolating the high-quality nascent RNAs from plant tissue is still challenging due to the presence of cell walls and other reasons. Several methods, such as CB-RNA-seq, pNET-seq, and GRO-seq, have been developed recently to detect nascent RNA and reveal plant-specific transcriptional features. In this study, we provided a detailed protocol for isolation of the chromatin-bound RNA from *A. thaliana* seedlings. Given the unique advantages of this approach, the CB-RNA extraction method is considered the most straightforward way for nascent RNA isolation. Firstly, the procedure is relatively simple, time-saving, and can be done with limited materials (One gram of seedlings are sufficient for most of applications). The entire CB-RNA isolation procedure can be done within 2–3 h from raw materials to purified RNAs. Secondly, the method is more economical compared with other parallel methods such as pNET-seq and GRO-seq given that CB-RNAs isolation does not involve immunoprecipitation steps that are time-consuming and expensive. Thirdly, once the CB-RNAs is obtained, it can be handled the same way as the total RNA, which is compatible with all sorts of downstream applications or sequencing library construction strategies. For example, the isolated CB-RNAs can be combined with specific downstream methods to assay 3’ end processing, RNA methylation, RNA editing, anti-sense RNA, circular RNA, and so on at the chromatin level. CB-RNA isolation method should be applicable in a wide range of plant species as illustrated successfully in Soybean [20]. However, extract CB-RNAs from seeds or the other tissues that are rich in secondary metabolites (such as lipid, starch…), extra steps might be needed to remove those distractors before better CB-RNA isolation.

There are also limitations for CB-RNAs isolation and sequencing. Firstly, our current protocol only recovers CB-RNAs above 200nt. This is due to the column purification of CB-RNA (with 200nt cut-off for the Qiagen RNA miniprep kit) and also the size selection in the Illumina RNA-seq library generation step. Thus, caution and adjustment of the protocol will be needed when the nascent RNAs below 200nt are of interest. Secondly, in association with the first point, the CB-RNAs is suitable for studying nascent RNA and its associated RNA-processing steps but less suitable for looking at the Pol II occupancy. For example, Pol II pauses at both 5ʹ and 3ʹ end of the gene in Arabidopsis [15], while the paused Pol IIs are likely associated with nascent RNA smaller than 200nt, which would not be resolved effectively in CB-RNA-seq. Indeed, both the 5ʹ and 3ʹ end pause of Pol II were not observed in our CB-RNA-seq data. For accurate Pol II profiling, NET-seq is recommended. Thirdly, the inactivation of endogenous RNAase during the CB-RNA isolation procedure is critical and sometimes can cause problem. Plant cells contain rich sources of endogenous RNase. In the current protocol, high concentrations of RNase inhibitors and yeast tRNAs are employed to buffer against endogenous RNase activity. In addition, the handling time from cell lysis to the urea washing steps needs to be minimized. Thus, we recommend only handling one or two samples simultaneously for these steps. After the UREA washing steps (Fig. 1A), all the samples could be handled together for the following RNA extraction.

Our data showed that a small fraction of full-length polyadenylated RNA (RNAf) is included in the isolated CB-RNAs (Fig. 5D). The incomplete splicing status, plus the observed cooperativity among different intron splicing in this RNA fraction, suggest these RNAs are most likely the RNA processing intermediates that are polyadenylated but not yet released from chromatin (Fig. 5D). Thus, they represent a fraction of RNAs that sit on chromatin but not the contamination during CB-RNAs purification. Thus, in most cases, it is unnecessary to remove these polyadenylated RNAs prior to sequencing library construction or qPCR-based analysis. However, the removal of polyadenylated RNA is needed when only the elongating RNA is of interest. The presence of polyadenylated but not fully spliced RNA on chromatin suggests there is no strict order between polyadenylation and splicing. In addition, gene splicing status might be monitored by a mechanism linked with Pol II at gene 3’ end such that transcripts with unspliced intron are not effectively released from chromatin even when it is polyadenylated. An exciting hypothesis that awaits to be explored in the future.

## Conclusions

We described a simple and robust method to enrich chromatin-bound RNAs that include nascent RNA undergone elongation by Pol II and polyadenylated RNA that has undergone RNA processing on chromatin. The isolated chromatin-bound RNAs are compatible with all sorts of downstream applications, such as the studying of co-transcriptional RNA-processing combined with next-generation sequencing.

## Methods

### Plant material and growth conditions

*Arabidopsis thaliana* (Col-0) seeds were sterilized in 10% sodium hypochlorite for 10 min and rinsed by sterile distilled water 3 to 4 times before being sown on 1/2 MS media. Seeds were stratified at 4 °C for 3 days and transferred to a growth chamber (Percival CU-36L5), 16 h light/8 h dark, 22 °C. 10-day-old seedlings were collected and snap-frozen in liquid N2 for the following chromatin-bound RNAs (CB-RNAs) extraction.

### Chemicals and reagents

Note that RNase-free chemicals and solution are critical for CB-RNA isolation.

#### Chemicals

Sucrose (Sigma S0389), Ficoll (Sigma F4375), Dextran T40 (Sigma 31389), HEPES (Sigma 54457), MgCl_2_ (Invitrogen AM9530G), Triton X-100 (Sigma T8787), DTT (Sigma 43,815), Proteinase inhibitor cocktail (Roche 11873580001), Glycerol (Invitrogen 15514-011), 1 M EDTA (Invitrogen 15575-038), 1 M Tris–HCl pH 7.5 (Invitrogen 15567-027), 5 M NaCl (Invitrogen AM9759), Urea (Sigma U5378), Tween-20 (Sigma P7949), Chloroform (Analytical grade), Ethanol (Analytical grade), Isopropyl alcohol (Analytical grade), Nuclease-free H_2_O (Invitrogen 10977-015).

#### Reagents

Yeast-tRNA (Roche 10109525001), RNase inhibitor (Promega N251A), RNaseZap (Invitrogen AM9780), TRIzol Reagent (Invitrogen 15596018), 3 M Sodium Acetate pH5.5 (Invitrogen AM9740), Perfect Start Green qPCR SuperMix (TransGene AQ601-04), RNeasy RNA purification Kit (Qiagen 74104), mRNA Capture Beads (Vazyme N401), Qubit RNA Assay Kit (Qubit Q32852), RNA secure Reagent (Ambion AM7006), Turbo DNase (Invitrogen AM2238), riboPOOLs (rRNA removal probes) (siTOOLs Biotech), Streptavidin magpoly beads (SMART lifesciences SM01710), Low Melting Point Agarose (Invitrogen 16520-050), 10 × TBE Buffer (Invitrogen 15581-044), DNA size Marker (DL1000, Takara 3591Q), NEBNext Ultra II Directional RNA Library Prep Kit for Illumina (NEB E7760).

#### Consumables

Phase-lock tube (Invitrogen A33248), 0.45 μm sterile filter (250 mL, BIOFIL FPE-414-250); 0.45 μm nylon membrane (Nalgene), Miracloth (Millipore 475855), 50 mL Plastic Funnel, Nuclease-free 1.5 mL, 2 mL and 0.2 mL microcentrifuge tubes; Nuclease-free 15 mL and 50 mL falcon tubes; Nuclease-free pipette tips.

#### Antibodies

anti-H3 (Abcam 1791), anti-Ser2P CTD Pol II (Active Motif 61083), anti-UBC (abcam, ab99002).

### Buffer compositions

For CB-RNA isolation:

#### Honda buffer

0.44 M sucrose, 1.25% (wt/vol) Ficoll, 2.5% (wt/vol) Dextran, 20 mM HEPES pH 7.4, 10 mM MgCl_2_, 0.5% Triton X-100, 2 mM DTT freshly added, 1 × cocktail freshly added (100 × cocktail stock solution: 2 tablet/ml Nuclease-free H_2_O), 1 × tRNA freshly added (100 × tRNA stock solution: 10 μg/μL), 1 μl RNase inhibitor was added in every 100 μL buffer. Adjust pH with 1 M KOH to 7.5. The solution is filtered through 0.45 μm PES membrane and can be kept at − 20 °C for up to 3 months.

#### Nuclei resuspension buffer

50% Glycerol, 0.5 mM EDTA, 25 mM Tris–HCl pH 7.5, 100 mM NaCl, 2 mM DTT freshly added, 5 × tRNA freshly added, 2 × cocktail freshly added, 2 μL/100 μL RNase inhibitor freshly added. Solution is filtered through 0.45 μm nylon membrane and can be kept at − 20 °C for up to 3 months.

#### Wash buffer

25 mM Tris–HCl pH 7.5, 300 mM NaCl, 1 M Urea, 0.5 mM EDTA, 1% Tween 20 Solution is filtered through 0.45 μm nylon membrane and can be kept at − 20 °C for up to 3 months.

For rRNA removal:

#### Hybridization buffer

10 mM Tris–HCl (pH 7.5); 1 mM EDTA; 2 M NaCl.

#### Beads resuspension buffer

0.1 M NaOH; 0.05 M NaCl.

#### Depletion buffer

10 mM Tris–HCl (pH 7.5); 1 mM EDTA; 1 M NaCl.

### Protocol for Chromatin-bound RNA extraction

#### Before start

- Use RNasezap to clean the bench and pipette before starting.
- Pre-cool the centrifuge to 4 °C.
- Prepare working solutions (e.g., Honda buffer 10 mL/sample; Nuclei resuspension buffer 800 μL/sample; Wash buffer 1.2 mL/sample) freshly before use.
- Given that endogenous RNAase can’t be inactivated thoroughly during the procedure, handling one sample each time is recommended.

### *Chromatin extraction (*~ *2 h)*

1. Take 1 g of Arabidopsis seedlings and grind them in liquid nitrogen to a fine powder.
2. Resuspend the ground powder with 8 mL pre-cooled Honda buffer and homogenize quickly by stirring with a 1 mL tip. Filter the solution through two layers of Miracloth into a new ice-cold 50 mL falcon tube. (Note: 2 mM DTT, 1 × tRNA solution, 80 μL RNase inhibitor, and 1 × proteinase inhibitor cocktail are added into 8 mL Honda buffer just before use).
3. Centrifuge the filtrate immediately at 4 °C, 3500*g* for 5 min.
4. Remove the supernatant, resuspend the pellet immediately with 1 mL pre-cooled Honda buffer, and transfer to a new ice-cold 2 mL tube. (Note: 2 mM DTT, 5 × tRNA solution, 10 μL RNase inhibitor, and 1 × proteinase inhibitor cocktail are added into 1 mL Honda buffer just before use).
5. Centrifuge immediately at 4 °C, 8000*g* for 1 min.
6. Remove the supernatant thoroughly and weigh the pellet.
7. Resuspend the pellet with 1 volume (w/v) of nuclei resuspension buffer. (e.g., resuspend 100 mg nuclei pellet with 100 μL nuclei resuspension buffer). Stir to mix the pellet and pipet up and down several times with an end-cut 200μL tip. (Note: 2 mM DTT, 5 × tRNA solution, 2 × proteinase inhibitor cocktail, and 1 μL RNase inhibitor in every 50 μL buffer are added into nuclei resuspension buffer just before use).
8. After homogenization, add 2 volumes of wash buffer into the above mixture, and pipette gently 20 times with an end-cut 1 mL tip to ensure samples are thoroughly mixed. (Note: Take ~ 1/10 resuspended nuclei at this step and snap frozen in liquid nitrogen if the nuclear fraction of RNA needs to be assessed by qPCR analysis.)
9. Store the mixture on ice for 1 min.
10. Centrifuge immediately at 4 °C, 8000*g* for 1 min. Remove the supernatant thoroughly. (Note: Keep the supernatant as wash I and snap frozen in liquid nitrogen if this RNA fraction needs to be assessed by qPCR analysis.)
11. Resuspend the pellet again with 1 volume of nuclei resuspension buffer. Stir to mix the pellet and pipette gently several times with an end-cut 200 μL tip.
12. After homogenization, immediately add 1 volume of wash buffer to the mixture and pipette up and down 20 times with an end-cut 1 mL tip to ensure samples are thoroughly mixed.
13. Store the mixture on ice for 1 min.
14. Centrifuge immediately at 4 °C, 12,000*g* for 2 min. Remove the supernatant thoroughly. (Note: Keep the supernatant as wash II and snap frozen in liquid nitrogen if this fraction of RNA needs to be accessed by qPCR analysis.)
15. Resulting chromatin can be snap frozen and stored at − 80 °C.

### Chromatin-bound RNA purification (~ 1.5 h)

16. Resuspend the pellet from step 15 (Chromatin-Bound fraction), step 8 (100 μL, nuclear fraction), step 10 (100 μL, wash I fraction), and step 14 (100 μL, wash II fraction) with 1 mL Trizol solution.
17. Vortex to mix thoroughly and stay at room temperature for 10 min.
18. Add 0.2 mL chloroform, vortex for 10 s, and leave at room temperature for 5 min.
19. Spin the phase-lock tube at 14,000*g* for 2 min at room temperature just before use. Transfer the solution from step 18 to a phase-lock tube. Centrifuge at 14,000*g*, 10 min at 4 °C.
20. Carefully transfer the supernatant into a new tube and add a 0.8 × volume of 100% ethanol.
21. Rotate to mix and centrifuge briefly at 600 g for a few seconds. Transfer the solution (including precipitation if any) to a Qiagen RNA Column. Centrifuge multiple times at 8000*g* room temperature for 1 min until all the solutions pass through.
22. Wash the column with 700 μL RW1 buffer, centrifuge at room temperature, 12,000*g* for 1 min.
23. Wash the column with 500 μL RPE buffer, centrifuge at room temperature, 12,000*g* for 1 min. Repeat once.
24. Change to a new 2 mL tube. Centrifuge at 12,000*g* for 2 min.
25. Change the column to a new 1.5 mL tube. Add 50 μL Nuclease-free H_2_O, stay on bench for 2–5 min, centrifuge 12,000*g* for 1 min to elute. Repeat once and obtain 100 μL eluted RNAs in total.
26. Measure the RNA concentration with a Nano-Drop machine.

### DNA contamination removal (~ 2.5 h)

27. Set up the following Turbo DNase I digestion reaction:
  RNA sample (2 to 5 μg): 100 μL
  10 × DNase I buffer: 12 μL
  RNase Inhibitor (40U/μL): 2 μL
  Turbo DNase I (2U/μL): 2 μL
  25 × RNA secure H_2_O: 4 μL
28. Incubate at 37 °C for 1 h.
29. Add 3.5 volumes of RLT buffer and 2.5 volumes of Ethanol to the sample from the last step and mix well. (Qiagen RNeasy Mini Kit)
30. Transfer the mixture to a Qiagen RNA Column and centrifuge at 10,000*g* for 30 s at room temperature. Perform multiple times until all the solutions pass through.
31. Discard the flowthrough. Add 500 μL RPE buffer to the column and centrifuge at 12,000*g* for 30 s at room temp. Repeat once and centrifuge at 14,000*g* for 2 min.
32. Transfer the column to a new 1.5 mL Tube.
33. Add 50 μL RNase-free water to column and still for 5 min. Then centrifuge at the 14,000*g* for 1 min. Repeat once.
34. Transfer 100 μL eluted RNA sample to a fresh 1.5 mL tube.
35. Set up the precipitation reaction as follow:
  RNA sample: 100 μL
  3M NaOAc: 10 μL
  Isopropanol: 110 μL
  Glycoblue: 1 μL
36. Incubate at − 80 °C for 30 min, and spin at 14,000*g* 4 °C for 15 min.
37. Wash the RNA pellet with 80% ethanol twice.
38. Remove any solution thoroughly with a pipette and dissolve the RNA pellet with 15 μL H_2_O.
39. Check the RNA concentration with Nanodrop and take 1 μL (≥ 10 ng) for RNA quality check by running it on a 2% (wt/vol) low melting agarose gel (1 × TBE gel with 1 × SYBR gold fluorescent dye added).
40. Take 500 ng RNA sample for qPCR analysis. Alternatively, the RNA sample can be subjected to poly(A) RNA removal and rRNA-depletion steps, followed by RNA-seq library construction. The isolated chromatin-bound RNAs can be stored at − 80 °C for several months.

### qPCR analysis to validate nascent RNA enrichment at individual genes

1. Set up the following reverse transcription reaction mix (10 μL):
  RNA sample (500 ng): 5.5 μL
  10 mM dNTPs: 0.5 μL
  Primer mix: 0.5 μL
  Heat the mixture at 65 °C for 5 min and place the tube on ice for at least 2 min. (The primer mix needs to be prepared according to the number of gene-specific primers used. 1 pmol of each primer is included for each reaction. A list of primers used in this study can be found in Table 2)
  Prepare the following as mix and add 3.5 μL into each reaction.
  5x M-MLV buffer: 2 μL
  M-MLV Enzyme (200U/μL): 0.5 μL
  RNase Inhibitor (40U/μL): 0.5 μL
  RNase-free water: 0.5 μL
2. Place the tube on a PCR thermocycler with the 105 °C heated lid and perform the PCR cycles as follows:
  42 °C 1 h, 80 °C 5 min, and hold at 4 °C.
3. Dilute the cDNA sample 40 times with nuclease-free water.
4. Set the following qPCR reaction mix (10 μL):
  cDNA (Diluted): 4 μL
  qPCR SuperMix: 5 μL
  10 μM F Primer: 0.5 μL
  10 μM R Primer: 0.5 μL
  (A list of primers used in this study can be found in Table 2)
5. Place the tube on a qPCR thermocycler and perform the PCR cycles as follows:
   95 °C 2 min; 95 °C 15 s, 60 °C 20 s, 72 °C 25 s, 45 cycles.
6. For qPCR analysis, the relative enrichment of CB-RNAs can be determined by comparing the ratio of unspliced RNA to spliced RNA (Fig. 2) in different fractions (total RNA, nuclear RNA, wash I, wash II, CB-RNA).

**Table 2:** Primers used in this study

Primer Name	Sequence 5ʹ to 3ʹ
AT1g17220-Intron6-Spliced-F	5ʹ TTGTTGCAGAGCTTCAAGAGT 3ʹ
AT1g17220-Intron6-Spliced-R	5ʹ CTTCTCCGCAAACAACAACA 3ʹ
AT1g17220-Intron6-UnSpliced-F	5ʹ TGCTTTGAAAGGGGAAAATG 3ʹ
AT1g17220-Intron6-UnSpliced-R	5ʹ CCGAAAACAGAAACGGAAAA 3ʹ
AT1g57820-Intron1-Spliced-F	5ʹ GCCTGAGAGACCTGTGACGAAA 3ʹ
AT1g57820-Intron1-Spliced-R	5ʹ TGGCAGCAACAAGAGACGAGTT 3ʹ
AT1g57820-Intron1-Unpliced-F	5’ TCTTGGGCTGATGGAAATCTTGTG 3ʹ
AT1g57820-Intron1-Unpliced-R	5ʹ CAGGAATTATGCTGCGGCATTTAC 3ʹ
AT2g15620-Intron2-Spliced-F	5ʹ TTTCACCAACTTGCCAAGAAAG 3ʹ
AT2g15620-Intron2-Spliced-R	5ʹ AATTGAATCCAAACCGTCCA 3ʹ
AT2g15620-Intron2-Spliced-F	5ʹ GAGTGAATGGTTATGCTAAGATGG 3ʹ
AT2g15620-Intron2-Spliced-R	5ʹ AGATCATGAGTCCCCACCAC 3ʹ
AT3g28860-Intron9-Spliced-F	5ʹ TCACATCCTTCATAGTCGCCTTCA 3ʹ
AT3g28860-Intron9-Spliced-R	5ʹ GTTACTGACTCCTTCACCAGCAATC 3ʹ
AT3g28860-Intron9-Unspliced-F	5ʹ GTGGCGACTTAGACCTTTGATTGT 3ʹ
AT3g28860-Intron9-Unspliced-R	5ʹ TGCATGAGCCTTAGCTGTGTCTCCAG 3ʹ
AT5g55220-Intron5-Spliced-F	5ʹ GGCGATGAGATAGATGCAAAA 3ʹ
AT5g55220-Intron5-Spliced-R	5ʹ GCTAGATCTCCCACCTGTAGC 3ʹ
AT5g55220-Intron5-Unspliced-F	5ʹ ATGTCCTAAAACCGTGTAGGTG 3ʹ
AT5g55220-Intron5-Unspliced-R	5ʹ AGCATCTGGAATCGCTTGA 3ʹ

### Removal of chromatin-bound polyadenylated RNA (optional for CB-RNA-seq)

1. Take out mRNA Capture Beads (Vazyme) from 4 °C and warm them to room temperature.
2. Take 8 μg chromatin-bound RNA sample. Dilute it to 50 μL with nuclease-free water in a nuclease-free 1.5 mL tube.
3. Gently mix mRNA Capture Beads and transfer 50 μL beads into the RNA sample. Mix with a pipette.
4. Denature the RNA by incubation at 65 °C for 5 min and place the tube on ice immediately.
5. Leave the sample at room temperature for 5 min.
6. Place on the magnet for 5 min to separate poly-A RNA from the rest of the CB-RNAs.
7. Take out the supernatant to a new 1.5 mL tube and set it aside.
8. Add 200 μL Beads Wash buffer to beads and mix with pipette thoroughly.
9. Place the tube on a magnet for 5 min and discard the supernatant.
10. Add 50 μL Tris Buffer to resuspend beads and mix with pipette thoroughly.
11. Incubate the tube at 80 °C for 2 min and set the tube at room temperature for 2 min.
12. Add 50 μL Beads Binding buffer and mix with pipette thoroughly. Incubate the tube at room temperature for 5 min.
13. Place the tube on a magnet for 5 min, transfer the supernatant to a new 1.5 mL tube, and mix with the sample in step 7.
14. Add 1/10 volume of 3 M NaOAc and 1 μL Glycoblue, and mix thoroughly.
15. Add 1 volume of isopropanol and mix thoroughly.
16. Incubate at − 80 °C for 20 min.
17. Centrifuge at 14,000*g* 4 °C for 10 min.
18. Remove supernatant and wash the pellet twice with 80% ethanol.
19. Centrifuge at 14,000*g* 4 °C for 5 min and discard the supernatant.
20. Dissolve the pellet in 13 μL of RNase-free water.

### Removal of rRNA from chromatin-bound RNA (for CB-RNA-seq)

- **Hybridization of riboPOOL probe to RNA**
  1. Set up the following reaction mix:
    RNA sample (2 μg CB-RNAs): 13 μL
    50 μm riboPOOL probe: 1 μL
    Hybridization buffer: 5 μL
    RNase inhibitor (40U/μL): 1 μL
  2. Vortex to mix and spin down the droplet.
  3. Incubate at 68 °C for 10 min to denature RNA.
  4. Allow cooling slowly to 37 °C for optimal hybridization. (By setting ramp rate at 3 °C/min)
- **Preparation of beads (Two steps of depletion)**
  5. Resuspend the Streptavidin magpoly beads by carefully vertexing the tube at medium speed.
  6. Transfer 60 μL of beads suspension per sample to a fresh tube. (To prepare multiple samples, aliquot beads suspension for up to 8 samples (i.e., 480 μL) in a single tube).
  7. Place the tube on a magnetic rack and wait for 1 min.
  8. Aspirate and discard all supernatant.
  9. Add 100 μL per sample of beads Resuspension buffer, and agitate the tube well to resuspend beads. Place on magnetic rack for 1 min, aspirate and discard supernatant.
  10. Repeat step 9.
  11. Resuspend beads in 80 μL depletion buffer per sample and divide the beads into 2 tubes.
- **Ribosomal RNA depletion**
  12. Briefly centrifuge the tube containing ~ 20 μL hybridized riboPOOL and CB-RNAs.
  13. Transfer 40 μL of the prepared beads (leave another 40 μL beads on the magnetic rack, wait for 1 min, and discard the supernatant) into the tube containing hybridized riboPOOL-RNA solution. Agitate the tube to resuspend well.
  14. Incubate the tube at 37 °C for 15 min (mix every 5 min), followed by a 50 °C incubation for 5 min.
  15. Spin briefly and place the tube on the magnet for 2 min then carefully transfer the supernatant to the other tube containing beads (without depletion buffer from step 13), repeat step 14.
  16. Place the tube on the magnet for 2 min and carefully transfer the supernatant to a new tube.
- **RNA clean-up**
  17. Add nuclease-free water to the rRNA-depleted RNA sample from the last step to bring the volume to 100 μL.
  18. Set up the following precipitation reaction:
    RNA sample: 100 μL
    10 mg/mL Glycoblue: 1 μL
    3M NaOAc: 11 μL
    100% ethanol: 280 μL
  19. Mix well, and incubate at − 20 °C for at least 1 h.
  20. Centrifuge at 14,000*g* 4 °C for 10 min.
  21. Remove supernatant and wash twice with 80% ethanol.
  22. Centrifuge at 14,000*g* 4 °C for 5 min and discard the supernatant.
  23. Air dry and dissolve the pellet in 12 μL of RNase-free water.
  24. Check the RNA concentration with Qubit RNA Assay Kit and take 50 ng for CB-RNAs library construction.

### Next-generation sequencing and bioinformatic analysis

Paired-end sequencing was performed on the bar-coded library using an Nova Seq 6000. The adapters, Ns and low-quality bases were removed from raw data using the Trimmomatic [28] package (version 0.39). Then, the reads shorter than 36 bp were also removed. And the clean reads were mapped to the TAIR10 genome using the HISAT2 (version 2.2.0) [29] with default parameters. The reads with more than one reported alignment were excluded. The mapped reads were sorted and indexed by SAMtools [30] (version 1.3.1). The Fragments Per Kilobase of exon model per Million mapped fragments (FPKMs) of each gene were calculated by StringTie [31] (version 2.1.4). Genes with FPKM ≥ 1 were used for the calculation of 5’ SS or 3’ SS ratio. And the calculation method was same as the previous study [18]. The structure of the longest transcript was used as representative gene structure. And the average SS ratio of all the introns represents the gene’s SS ratio. For metagene profiling, TPM was calculated with a 5-bp sliding window, and profiles were visualized using plotProfile in deepTools [32]. Although a strand-specific RNA-seq library was generated with commercial kit from NEB (E7760), it is worth noting that for the Arabidopsis genome, antisense transcription is quite rare. Hence for make metagene profiling, any reads that belongs to antisense transcription for genes across the genome were kept. The stand-specific profile (with antisense transcription filtered out) looks similar to non- strand-specific profile (data not shown). The detailed pipeline of bioinformatic analysis can be find in Additional file 1.

## Supplementary Information

**Additional file 1: **Bioinformatic pipeline.

**Additional file 2: Fig. S1.** The relationship between co-transcriptional splicing (CTS) efficiency and intron order in RNAe,RNAf, RNAe+RNAf and mRNA.

## Data Availability

Bioinformatic pipeline and scripts used in this study can be downloaded from https://github.com/flzh628/cb-RNA-seq. The raw sequencing data and processed files for CB-RNA-seq data generated in this study are available in the Gene Expression Omnibus (GEO) database under accession number GSE213583.
