# Supplementary material for: A simple and robust method for isolating and analyzing chromatin-bound RNAs in Arabidopsis
Source: Plant Methods. 2022 Dec 12;18:135. doi: 10.1186/s13007-022-00967-y (PMC9743689; doi:10.1186/s13007-022-00967-y)
Supplement: Supplementary file 1 — Additional file 1: Bioinformatic pipeline. [file 13007_2022_967_MOESM1_ESM.docx]

**Bioinformatic pipeline**

1. Preparatory work. Download the scripts and necessary files from GitHub repository: https://github.com/flzh628/cb-RNA-seq. TAIR10 genome sequence and annotation files were downloaded from the Gramene website (https://ensembl.gramene.org/Arabidopsis_thaliana/Info/Index).

2. Quality control of raw data. The adapters, Ns and low-quality bases were removed from raw data using the Trimmomatic [26] package (version 0.39). And the reads shorter than 36 bp were also removed.

$ java -jar /path/to/Trimmomatic/trimmomatic-0.39.jar PE -phred33 -threads 64 Read1.fastq.gz Read2.fastq.gz Read1.paired.fq.gz Read1.unpaired.fq.gz Read2.paired.fq.gz Read2.unpaired.fq.gz ILLUMINACLIP:/path/to/Trimmomatic/adapters/TruSeq3-PE-2.fa:2:30:10 LEADING:3 TRAILING:3 SLIDINGWINDOW:4:15 MINLEN:36

3. Read mapping. The clean reads were mapped to the TAIR10 genome using the HISAT2 [27] (version 2.2.0) with default parameters. The reads with more than one reported alignment were excluded. The mapped reads were sorted and indexed by SAMtools [28] (version 1.3.1).

$ hisat2 -p 64 --dta -x /path/to/TAIR10/index/TAIR10 -1 Read1.paired.fq.gz -2 Read2.paired.fq.gz -S Sample.sam

$ less Sample.sam | grep '\bNH:i:1\b' | samtools view -T /path/to/TAIR10/TAIR10.fa -h - | samtools view -bS -@ 32 - > Sample.uniq.bam

$ samtools sort -@ 32 -O bam -o Sample.sort.bam -T temp Sample.uniq.bam

$ rm Sample.uniq.bam && mv Sample.sort.bam Sample.uniq.bam

$ samtools index Sample.uniq.bam

4. Quantification of gene expression. The Fragments Per Kilobase of exon model per Million mapped fragments (FPKM) of each gene were calculated by StringTie [29] (version 2.1.4).

$ stringtie -G TAIR10.gtf -o Sample.uniq.gtf -p 64 -e -b ballgown_out_ Sample -A Sample.uniq.gene.abundance Sample.uniq.bam

$ echo “LOC_name Sample.uniq.bam” > Sample.uniq.gene.FPKM

$ tail -n +2 Sample.uniq.gene.abundance | awk ‘{print $1”\t”$(NF-1)}’ >> Sample.uniq.gene.FPKM

5. The calculation of SS ratio. Genes with FPKM ≥ 1 were used for the calculation of 5’ SS or 3’ SS ratio. And the calculation method was same as the previous study [18]. The structure of the longest transcript was used as representative gene structure.

For one sample:

$ python IntronSpliceRatio_final.py --inputs Sample.uniq.bam --intron TAIR10.LongRNA.intron --output Sample.intron.SS-ratio --ex_chr ChrC,ChrM --geneflt Sample.uniq.gene.FPKM

For Multiple samples:

$ python IntronSpliceRatio_final.py --inputs Sample1.uniq.bam,Sample2.uniq.bam --intron TAIR10.LongRNA.intron --output Sample1_Sample2.intron.SS-ratio --ex_chr ChrC,ChrM --geneflt Sample1_Sample2.uniq.gene.FPKM

6. Downstream analyses. Downstream analyses included identifications of differentially cleaved introns, genes, and correlation analysis between SS ratio and intron number of gene. And the average SS ratio of all the introns represents the gene’s SS ratio.

$ python intron_SS_ratio_diff.py --ss_res Sample1_Sample2.intron.SS-ratio --tot_Col 10000000(# total read number of Sample1) --tot_mut 11000000(# total read number of Sample2) --names Sample1,Sample2

$ python intronSS_2_GeneLevel.py --inSS Sample1_Sample2.intron.SS-ratio --prefix Sample1_Sample2

$ python gene_SS_ratio_diff.py Sample1_Sample2.gene.SS-ratio --tot_Col 10000000(# total read number of Sample1) --tot_mut 11000000(# total read number of Sample2) --names Sample1,Sample2

$ awk ‘{print $1”\t”$7}’ Sample1_Sample2.gene.SS-ratio >Sample1.gene.SS5-ratio

$ python ssRatio_intronNum.py --pcg TAIR10.protein_coding_genes --intronNum TAIR10.LongRNA.intron_num --ratio Sample1.gene.SS5-ratio --prefix Sample1 --mark SS5
