# Supplementary material for: A simple and robust method for isolating and analyzing chromatin-bound RNAs in Arabidopsis
Source: Plant Methods. 2022 Dec 12;18:135. doi: 10.1186/s13007-022-00967-y (PMC9743689; doi:10.1186/s13007-022-00967-y)
Supplement: Supplementary file 2 — Additional file 2: Fig. S1. The relationship between co-transcriptional splicing (CTS) efficiency and intron order in RNAe,RNAf, RNAe+RNAf and mRNA. [file 13007_2022_967_MOESM2_ESM.pdf]

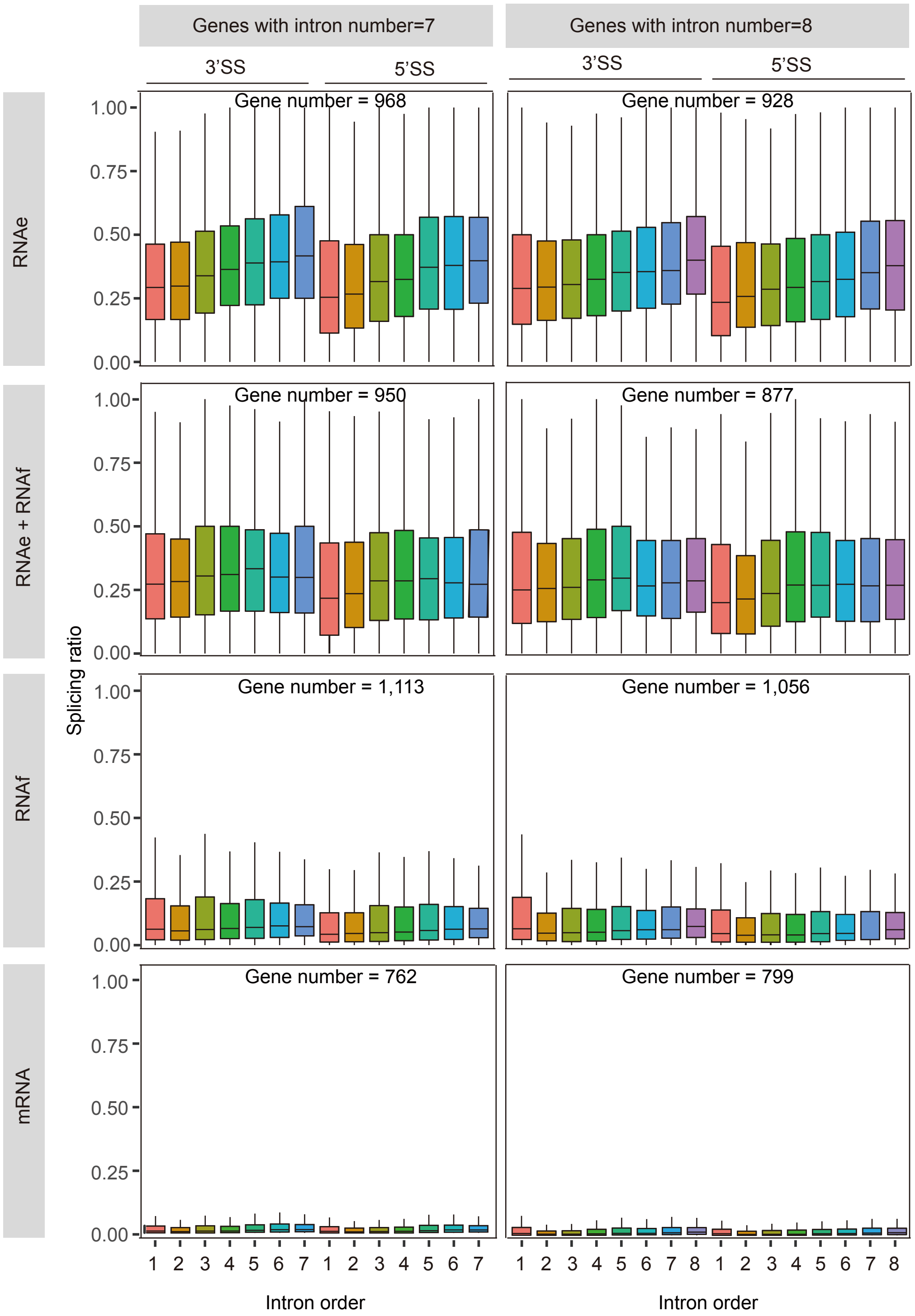

FigureS1. The relationship between co-transcriptional splicing (CTS) efficiency and intron order in RNAe, RNAf, RNAe+RNAf and mRNA.

The genes with length between 2k bp to 5k bp, containing 7 and 8 introns were selected as representitives. X-axis indicates the the intron order from 1st to 7th and 1st to 8th. The analyzed gene number was indicated at the top of each box.
